# Single nucleotide genome recognition and selective bacterial lysis using synthetic phages loaded with CRISPR-Cas12f1-truncated *sgRNA*

**DOI:** 10.71150/jm.2501012

**Published:** 2025-02-27

**Authors:** Ho Joung Lee, Song Hee Jeong, Sang Jun Lee

**Affiliations:** Department of Systems Biotechnology and Institute of Microbiomics, Chung-Ang University, Anseong 17546, Republic of Korea

**Keywords:** bacteriophage, CRISPR-Cas, single base mismatch, bacterial control

## Abstract

Phage specificity primarily relies on host cell-surface receptors. However, integrating *cas* genes and guide RNAs into phage genomes could enhance their target specificity and regulatory effects. In this study, we developed a CRISPR-Cas12f1 system-equipped bacteriophage λ model capable of detecting *Escherichia coli* target genes. We demonstrated that synthetic λ phages carrying Cas12f1-sgRNA can effectively prevent lysogen formation. Furthermore, we showcased that truncating the 3'-end of sgRNA enables precise identification of single-nucleotide variations in the host genome. Moreover, infecting *E. coli* strains carrying various *stx2* gene subtypes encoding Shiga toxin with bacteriophages harboring Cas12f1 and truncated sgRNAs resulted in the targeted elimination of strains with matching subtype genes. These findings underscore the ability of phages equipped with the CRISPR-Cas12f1 system to precisely control microbial hosts by recognizing genomic sequences with high resolution.

## Introduction

Bacteriophages are crucial for microbial regulation, offering alternatives to antibiotics due to their ability to kill host microbes (Rogovski et al., 2021). However, narrow host ranges and lysogeny formation can limit their efficacy (Lin et al., 2022). Nevertheless, advances in phage engineering have enabled the creation of synthetic phages with enhanced applications for microbial regulation (Jia et al., 2023; Lobocka et al., 2021). For instance, synthetic phages with altered host ranges can be produced using cell-free in vitro phage rebooting technology (Liang et al., 2022), and mutagenesis of phage tail fibers can modify host specificity (Yehl et al., 2019).

CRISPR-Cas systems are pivotal for genome editing in bacteriophages because they facilitate easy alteration of DNA specificity through guide RNA modules (Sternberg and Doudna, 2015). Duong et al. (2020) engineered a light-emitting synthetic phage by inserting the luciferase-encoding gene into the outer capsid protein gene of T4 phage using CRISPR-Cas9. Guan et al. (2022) used CRISPR-Cas13a to label the coat protein of the ФKZ jumbo phage with a fluorescent tag. Furthermore, CRISPR-Cas9 has been employed to invert the direction of the repressor *cI* gene in the temperate phage λ, converting it into an obligate lytic phage (Lee et al., 2022).

Additionally, phages can be used to deliver CRISPR-Cas systems to host bacteria. Nethery et al. (2022) developed a system that specifically edits *Escherichia coli* in a simulated co-culture of three microbial species using a cytosine base editor-equipped λ phage. Yosef et al. (2015) developed a system in which a Type I-E CRISPR-loaded λ synthetic phage removes antibiotic resistance plasmids from lysogenic strains, rendering pathogens susceptible to antibiotics.

Recently, the CRISPR-Cas system has been repurposed to specifically control target microbes. For instance, *cas9-sgRNA* genes were transferred from *E. coli* to *Salmonella enterica* through conjugation, resulting in the selective killing of *S. enterica* (Hamilton et al., 2019). M13-phagemids carrying the *cas9* gene (Lam et al., 2021) or non-replicating phages with inserted *cas9* genes (Mitsunaka et al., 2022) were engineered to specifically kill *E. coli* strains harboring target genes in the genome. Selle et al. (2020) reported that synthetic phages, with CRISPR arrays incorporated into the genome of ϕCD24-2 phages and key lysogeny genes removed, specifically killed *Clostridium difficile* and inhibited lysogeny in mouse colonic tissues.

Efforts have been made to enhance regulatory effects by incorporating *cas* genes and guide RNAs into phage genomes. Park et al. (Park et al., 2017; Cobb et al., 2019) reported that integrating the *cas9* gene into the genome of the temperate φ SaBov phage enhanced the killing effect of *Staphylococcus aureus* in mouse skin and rat osteomyelitis models. Jin et al. (2022) demonstrated that incorporating a 9.5 kb CRISPR-Cas3 cascade system into the λ genome would enable the specific control of enterohemorrhagic *E. coli* (EHEC) in an EHEC-infected mouse model. Gencay et al. (2024) incorporated a Type I-E CRISPR-Cas system into phage genomes to achieve phage-mediated specific killing of *E. coli* in the mouse gut environment. Previous studies have focused on regulating host bacteria by targeting specific genes. However, these methods often struggle to selectively control harmful bacterial strains that arise due to nucleotide variations, potentially affecting innocent cells.

In this study, we explored the potential for precisely controlling and suppressing lysogenic strain emergence by integrating the Cas12f1 system into the λ phage genome. With its compact size—approximately one-third that of commonly used Cas enzymes like Cas9 and Cas12a—Cas12f1 offers a significant advantage in facilitating seamless genomic incorporation (Kim et al., 2021b). Leveraging the single-base resolution of truncated guide RNAs obtained from previous studies (Lee et al., 2023), we aimed to identify and control target host bacteria based on single-base variations and genotypes in pathogenic microbe-like strain models using Cas12f1-loaded phages. Finally, based on the experimental results, we explored the prospects of precise microbial genome recognition and discussed control methods utilizing CRISPR-loaded phages.

## Materials and Methods

### Strains and cultivation

The *E. coli* strains used in this study are listed in Table S1. These strains were cultured in LB broth at 30°C or 37°C, depending on the specific requirements of each strain. To produce competent cells, the *E. coli* strains were cultured overnight at 30°C and then inoculated into LB broth at a final volume of 1%. The cultures were maintained at 30°C until the OD_600_ reached 0.4, at which point they were harvested. Strains MG1655 and HL051 harboring the pKD46 plasmid with λ red recombinase were further cultured for 3 h with a 1 mM final concentration of L-arabinose. Subsequently, the cultures were washed twice with 10% glycerol and aliquoted into 50 μl volumes for storage at -80°C. Depending on the selection marker of the plasmid or genetic cassette, ampicillin, kanamycin, chloramphenicol, and spectinomycin were added to the media at final concentrations of 50, 25, 12.5, and 75 μg/ml, respectively.

### Lysogeny construction

The HL051 strain (λ-lysogenic MG1655) was generated as follows: 150 μl of an MG1655 overnight culture was added to 15 ml of 0.6% soft agar supplemented with CaCl_2_ (5 mM) and MgSO_4_ (10 mM), mixed, and overlaid onto an LB agar plate (90 mm diameter). After air-drying for 20 min, 4 μl of λ *cI^857^* phage lysate was spotted on the agar, and the plate was incubated at 24°C for a week. Colonies from turbid spots were isolated, and lysogenic strain formation was confirmed using PCR with the primer pair (attB_F + λint_R) targeting the bacterial and phage genomes. Oligonucleotides for PCR are listed in Table S2.

### Host engineering

*E. coli* HL059 (MG1655 *galK* ^504^AT) was edited using CRISPR-Cas9, and then the *cas9* gene from the arabinose operon was removed via P1 transduction and the original *araBAD* operon was reintroduced. *E. coli* HL066 (MG1655 Δ*galK*) was generated by introducing the Δ*galK* mutation from the Keio collection JW0740 strain into MG1655 through P1 transduction (Baba et al., 2006). To produce an MG1655 strain carrying the *stx2* gene, the *stx2a* gene was amplified using genomic DNA from ATCC 43895 as a template, and the *stx2a*-CmR cassette was constructed and inserted by replacing the *srlAEBD* operon in the MG1655 genome. The *stx2a*-CmR cassette was inserted into the L-arabinose-induced MG1655 cells harboring the pKD46 plasmid through electroporation using a 0.1 cm cuvette at 25 µF, 200 Ω, and 1.8 kV. All subsequent electroporations were performed under the same conditions. After electroporation, 950 µl of the SOC medium was added to the cells, which were then allowed to recover at 30°C for 1 h and subsequently spread on chloramphenicol-containing LB agar and incubated at 37°C. The grown colonies were confirmed using PCR and further verified through Sanger sequencing to ensure that the generated HL085 strain carried the *stx2a*-CmR cassette. Nucleotide sequences for *stx2* subtypes were referenced from the following NCBI accession numbers: *stx2a* (CP008957) and *stx2g* (AY286000). Genomic DNA from the HL085 strain was used as a template for overlap PCR to amplify the *stx2g*-CmR cassette, and HL086 (MG1655-*stx2g*) was produced in the same manner as the HL085 (MG1655-*stx2a*) strain.

### *cas12f1* integration

To insert the *cas12f1* gene into the *b2* region of the λ phage genome from which the genes *ea59*, *ea47*, and *ea31* were deleted, a *cas12f1*-CmR cassette was constructed. The *P_rpsL_*-*cas12f1* fragment was generated using p15a-AsCas12f-apmR (a gift from Quanjiang Ji; Addgene plasmid #171610) as a template. The CmR cassette was amplified using genomic DNA from HK1020 as a template (Kim et al., 2021a). An overlapping PCR was performed to create the *P_rpsL_*-*cas12f1*-CmR cassette, which was then electroporated into L-arabinose-induced HL051 (λ *cI^857^* lysogen) cells harboring the pKD46 plasmid. The electroporated cells were spread on chloramphenicol-containing LB agar plates and incubated at 30°C. The resulting colonies were initially verified using PCR and further confirmed through Sanger sequencing to ensure the insertion of the *cas12f1*-CmR cassette into the *b2* region of the λ prophage genome in the finally constructed HL062 strain (λ *cI^857^* lysogen carrying *cas12f1* gene).

### *cas12f1*-*sgRNA* integration

Using the pHL267 plasmid (Lee et al., 2023) as a template, the *sgRNA* gene was amplified with the primer pair sgRNA_sacI_2F_N + KmR_sgRNA_N2_R. A 667 bp fragment of the *cas12f1* gene was amplified using genomic DNA from the HL061 strain as a template (Lee et al., 2023). The KmR cassette was amplified using genomic DNA from the HL002 strain and the primer pair sgRNA_KmR_N2_F + b2_KmR_int_R. An overlapping PCR was performed to construct a *cas12f1* (667 bp)-*sgRNA*-KmR cassette, which was then electroporated into the HL080 strain (λ *cI^857^* lysogen carrying the *cas12f1* gene and Δ*galK* mutation). The electroporated cells were spread on a kanamycin-containing LB agar plate and incubated at 30°C. Colony PCR and Sanger sequencing were performed to confirm the construction of the HL081 strain (λ *cI^857^* lysogen carrying the *cas12f1* gene and the *sgRNA* targeting *galK*). Subsequently, synthetic phages with a modified *sgRNA* target recognition sequence (TRS) within the prophage genome were constructed using overlapping PCR with the HL081 genome as a template.

### Spotting assay

Various phage-lysogenic cells were cultured overnight at 30°C. The supernatant from these cultures was spread on soft agar to yield phage plaques. An MG1655 overnight culture was inoculated into LB medium supplemented with CaCl_2_ (5 mM) and MgSO_4_ (10 mM) and cultured at 30°C with shaking at 180 rpm until the OD_600_ reached 0.4. Subsequently, phage plaques were added to these bacterial cultures, which were then incubated at 42°C until complete lysis occurred. The supernatant obtained after centrifugation (3,000 rpm, 4°C, 30 min), containing the phage lysates, was used to quantify plaque-forming units (pfu) and then stored at 4°C after adding 0.1% of CHCl_3_. To confirm the infectivity of the engineered phages and their ability to form clear or turbid spots, a spotting assay was performed. Specifically, 150 µl of each host strain’s overnight culture was added to 15 ml of 0.6% soft agar supplemented with CaCl_2_ (5 mM) and MgSO_4_ (10 mM), mixed, and overlaid on an LB agar plate (90 mm diameter). Then, 4 µl of phage lysates (~10^9^ pfu/ml) were spotted on the soft agar plates. The plates were incubated for 24 h at 30°C and 16 h at 37°C, respectively, depending on the specific requirements of each strain.

### Broth culture

To measure growth, single colonies of each host strain were grown overnight as starter cultures in LB broth at 30°C or 37°C with shaking at 180 rpm. For the main culture, 200 ml of LB broth in a 1 L flask was inoculated with the overnight culture to a final concentration of 1% and then incubated at 30°C or 37°C at 180 rpm until the OD_600_ reached 0.4. Subsequently, 49 ml of this culture was transferred into each 125 ml disposable flask. To achieve a multiplicity of infection (MOI) of 0.1, 1 ml of phage lysate was diluted and added to each flask, which was then incubated at 30°C or 37°C. The OD_600_ was measured using a spectrophotometer (Biochrom Libra S70, Harvard Bioscience, Inc., USA). Nineteen hours after phage addition, each culture was plated on LB agar plates and incubated at 30°C for 18 h. The resulting colonies were streaked onto kanamycin-containing LB plates to obtain single colonies. The presence of lysogeny in the obtained single colonies was confirmed using PCR with the primer pairs attB_F + λint_R and attB_F + bioB_R. 

## Results

### Lysogenization of bacteriophage λ in *E. coli*

**Fig. 1.**
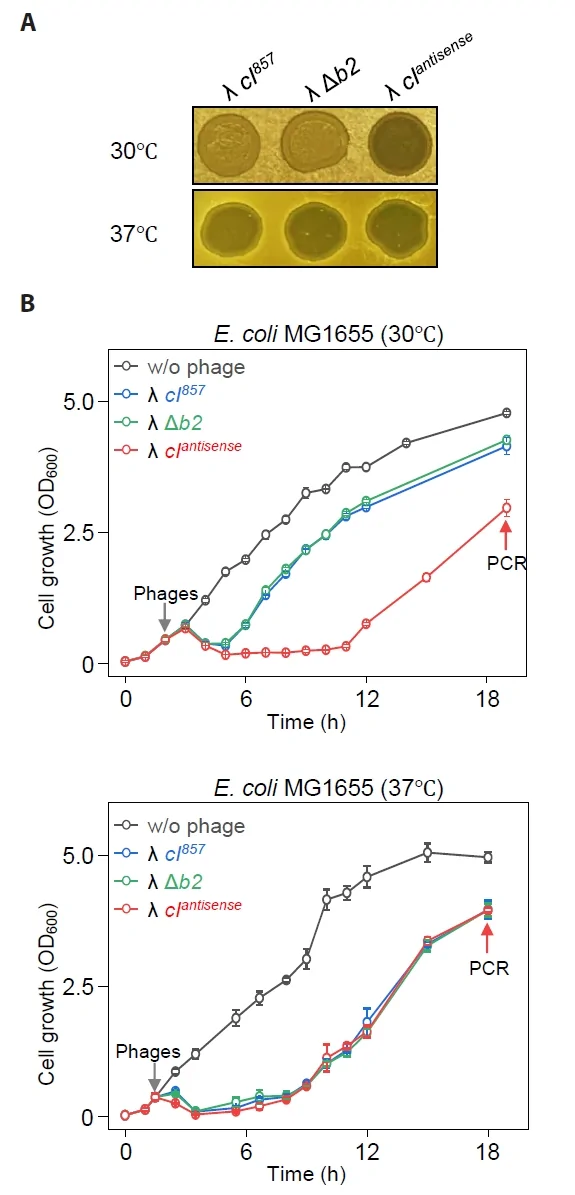
Growth of *E. coli* MG1655 cells infected with bacteriophages λ *cI^857^*, λ Δ*b2*, and λ *cI^antisense^* in solid and liquid media at 30°C and 37°C. (A) Spotting assay of bacteriophages on soft agar with MG1655 cells. Turbid and clear spots represent lysogen formation and lytic cell death, respectively. (B) Growth of phage-infected cells in flask cultures at different temperatures. Gray arrows indicate the time of phage infection, and red arrows indicate the time points of sample collection for PCR. The growth measurement results represent the average values obtained from three independent cultures.

We compared the turbidity of spots formed on soft agar plates inoculated with host MG1655 and various phages: λ *cI^857^* (George et al., 1987), λ Δ*b2* (with the non-essential gene removed), and the synthetic lytic phage λ *cI^antisense^* (Lee et al., 2022). The infection of λ *cI^857^* and λ Δ*b2* predominantly follows the lysogenic cycle at 30°C but shifts to the lytic cycle at 37°C. In contrast, λ *cI^antisense^* exclusively undergoes the lytic cycle, regardless of temperature conditions. The results showed that λ *cI^857^* formed turbid spots at 30°C and clear spots at 37°C. This indicates that at 30°C, lysogeny occurs, but at 37°C, the heat-labile nature of the CI^857^ repressor leads to its inactivation, triggering the λ lytic cycle (Fig. 1A). In contrast, the synthetic lytic phage λ *cI^antisense^* formed clear spots at all temperatures, as expected. Furthermore, λ Δ*b2* showed results similar to λ *cI^857^*, forming turbid spots at 30°C and clear spots at 37°C, suggesting that the removal of the *b2* region does not affect the phage life cycle.

Subsequently, we infected liquid cultures of host MG1655 with these phages at 30°C and 37°C and measured growth to compare with the results observed in solid media. When MG1655 was infected with the lytic phage λ *cI^antisense^* at 30°C, complete lysis occurred by 3 h, and growth resumed at the 12 h point on the graph, reaching an OD_600_ value of 3.0 by 19 h (Fig. 1B). Infections with λ *cI^857^* and λ Δ*b2* resulted in cell lysis, but cells began to regrow at 4 h post-infection, reaching an OD_600_ value of 4.2 by 19 h. Cells that regrew at the 19 h point (Fig. 1B) were no longer infected with λ *cI^857^*, and PCR results confirmed that the cells surviving λ *cI^antisense^* infection were phage-insensitive mutants. However, cells infected with λ *cI^857^* and λ Δ*b2* were confirmed to be lysogenic (Fig. S1). When MG1655 was cultured at 37°C and separately infected with λ *cI^857^*, λ Δ*b2*, and λ *cI^antisense^*, the patterns of host regrowth after lysis were the same. At 37°C, none of the regrown cells were infected with λ *cI^857^*, and PCR results confirmed that they were phage-insensitive mutant cells (Fig. S1).

These results demonstrate that λ *cI^857^*forms lysogens at 30°C but fails to do so at 37°C under both solid and liquid culture conditions. Additionally, the removal of the non-essential *b2* region in λ Δ*b2* does not substantially impact the ability of the phage to infect hosts and form lysogens at 30°C.

### Complete lysis of *E. coli* by bacteriophage λ with Cas12f1 nuclease

**Fig. 2.**
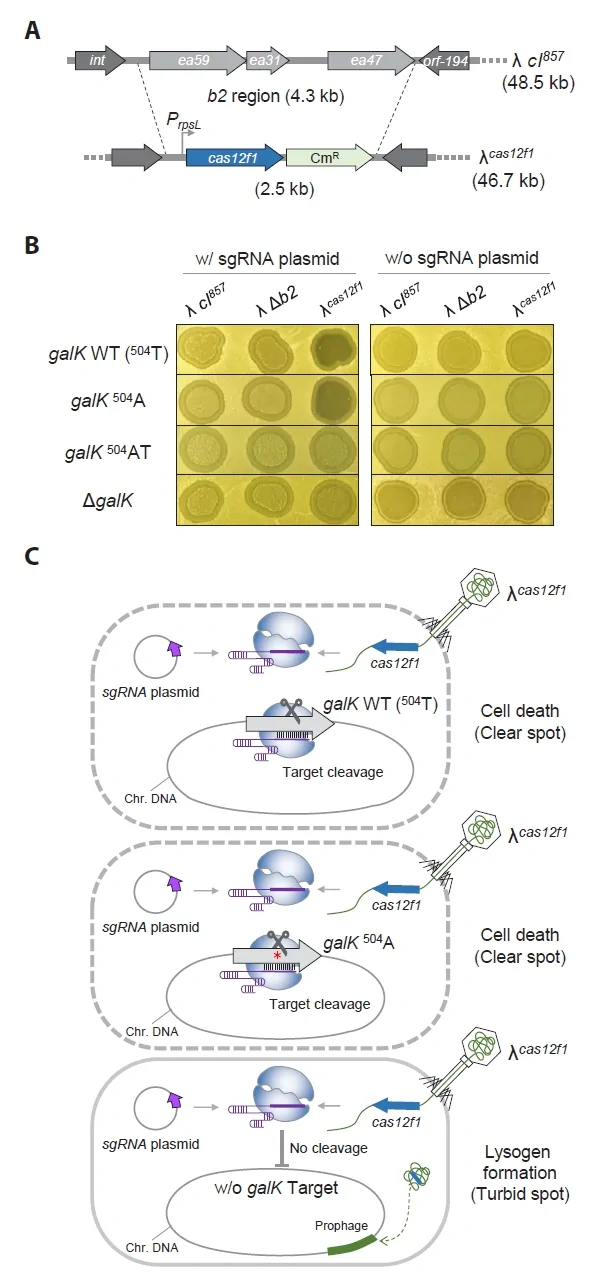
Genomic target recognition and complete cell lysis by phage λ carrying Cas12f1 nuclease. (A) Construction of λ*^cas12f1^*. The *cas12f1* gene was inserted into the *b2* region of the λ prophage genome via homologous recombination. (B) Spotting assay of λ*^cas12f1^*. Various *E. coli* MG1655 cells with different *galK* genotypes (*galK* WT, *galK* ^504^A, *galK* ^504^AT, and Δ*galK*) were tested. The cells were either transformed with or without a *galK*-targeting sgRNA plasmid. The phages λ *cI^857^*, λ Δ*b2*, and λ*^cas12f1^* were spotted on soft agar containing a culture of transformed cells and incubated at 30°C for 16 h. (C) Proposed mechanism of cell death. The Cas12f1 nuclease is delivered by the external phage λ carrying the *cas12f1* gene, while the sgRNA is supplied by a plasmid within the host cell. The Cas12f1-sgRNA complex cleaves host genomic DNA when the target sequence is present, causing cell death. If the target sequence is mismatched (indicated by a red asterisk) or absent, cleavage does not occur, allowing the host cell to survive and potentially enter lysogeny. This ensures that only target cells are lysed, while non-target cells remain viable.

We constructed the λ*^cas12f1^* phage by inserting the *cas12f1* gene at the location from which the *b2* region was removed from the genome (Fig. 2A). To test the functionality of this engineered phage, we engineered *E. coli* cells with various *galK* target sequences—*galK* ^504^A, *galK* ^504^AT, and Δ*galK* (Fig. S2)—and observed the turbidity of spots formed by the λ*^cas12f1^* phage on soft agar at 30°C, depending on the presence or absence of a *galK*-targeting sgRNA plasmid.

In cells harboring the sgRNA plasmid, clear spots were formed when the λ*^cas12f1^* phage was spotted on *galK* WT and *galK* ^504^A cells, while turbid spots were formed on *galK* ^504^AT and Δ*galK* cells (Fig. 2B). All cells without the sgRNA plasmid formed turbid spots. These results indicate that the expressed Cas12f1 and the sgRNA from the host’s sgRNA plasmid form a Cas12f1-sgRNA complex that effectively recognizes and cleaves the *galK* WT and *galK* ^504^A targets, causing cell death and clear spot formation. However, in the presence of two nucleotide (nt) mismatches (*galK* ^504^AT) or the absence of the *galK* target (Δ*galK*), the Cas12f1-sgRNA complex fails to cleave the genome, preventing cell death and allowing lysogeny to form, resulting in turbid spots (Fig. 2C).

**Fig. 3.**
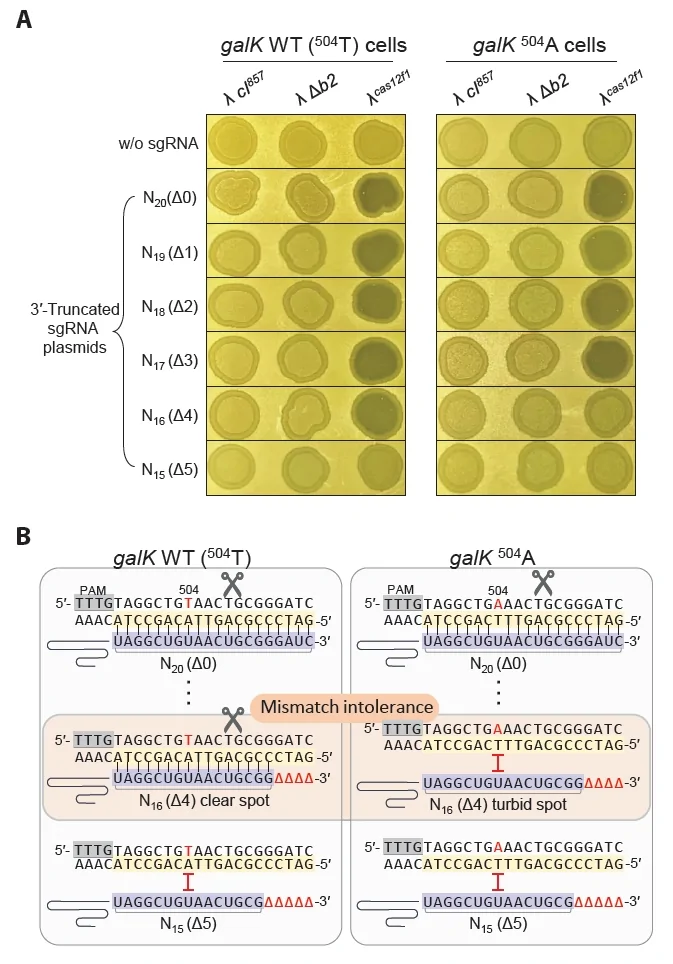
Recognition of single-nucleotide variations in the genome leveraging the mismatch intolerance of the 3ʹ-end truncated sgRNAs and phage λ-supplied Cas12f1 nuclease. (A) Spotting assay of λ*^cas12f1^*. MG1655 *galK* WT and ^504^A cells carrying different lengths of truncated sgRNAs were tested. The phages λ *cI^857^*, λ Δ*b2*, and λ*^cas12f1^* were spotted onto soft agar containing a culture of transformed *galK* WT and ^504^A cells and incubated at 30°C for 16 h. The terms Δ0–Δ5 indicate the number of 3′-end truncations in the sgRNA, and N_20_–N_15_ denotes the length of the sgRNA target recognition sequence (TRS). (B) Mismatch intolerance. The complex formed by the 3′-end truncated sgRNAs and the Cas12f1 nuclease can discriminate single-nucleotide variations in the genomic target. 504 refers to the nucleotide position within the *galK* structural gene. Red nucleotides T or A indicate variations at the 504th nucleotide position. Black vertical lines represent the base pairing between the non-PAM strand of the target DNA and the sgRNA. The scissor icon indicates that the Cas12f1-sgRNA complex recognizes and cleaves the target DNA. The Δ symbol denotes truncated nucleotides in the sgRNA. The red Ⅰ symbol indicates that the target DNA sequence is not recognized by the sgRNA as a cleavage target.

Next, to distinguish between *galK* WT and *galK* ^504^A, which differ by a single-nucleotide, we tested the effectiveness of a 3′-end truncated sgRNA approach (Lee et al., 2023) to overcome the mismatch tolerance of the Cas12f1 system. We performed the sameλ *^cas12f1^* phage spotting assay using sgRNAs with TRS lengths ranging from 20 nt (Δ0) to 15 nt (Δ5). When *galK* WT cells harboring Δ0 to Δ4 nt sgRNA plasmids were infected with the λ*^cas12f1^* phage, clear spots were formed (Fig. 3A). However, in *galK* ^504^A cells, clear spots were only formed with Δ0 to Δ3 nt sgRNA plasmids, while Δ4 nt sgRNA plasmid-bearing cells formed turbid spots.

When the phage spotting assay was performed at 37°C, clear spots were formed on all plates, regardless of the strain or phage type (Fig. S3). These results demonstrate that when host cells are infected with a Cas12f1-bearing bacteriophage, truncation of the sgRNA enables the differentiation of single-nucleotide variations in target genes (Fig. 3B) and allows for control over host cell lysogen formation and cell death.

### Genomic DNA sequence-specific lysis of *E. coli* via λ phage-mediated delivery of Cas12f1 and truncated sgRNAs

**Fig. 4.**
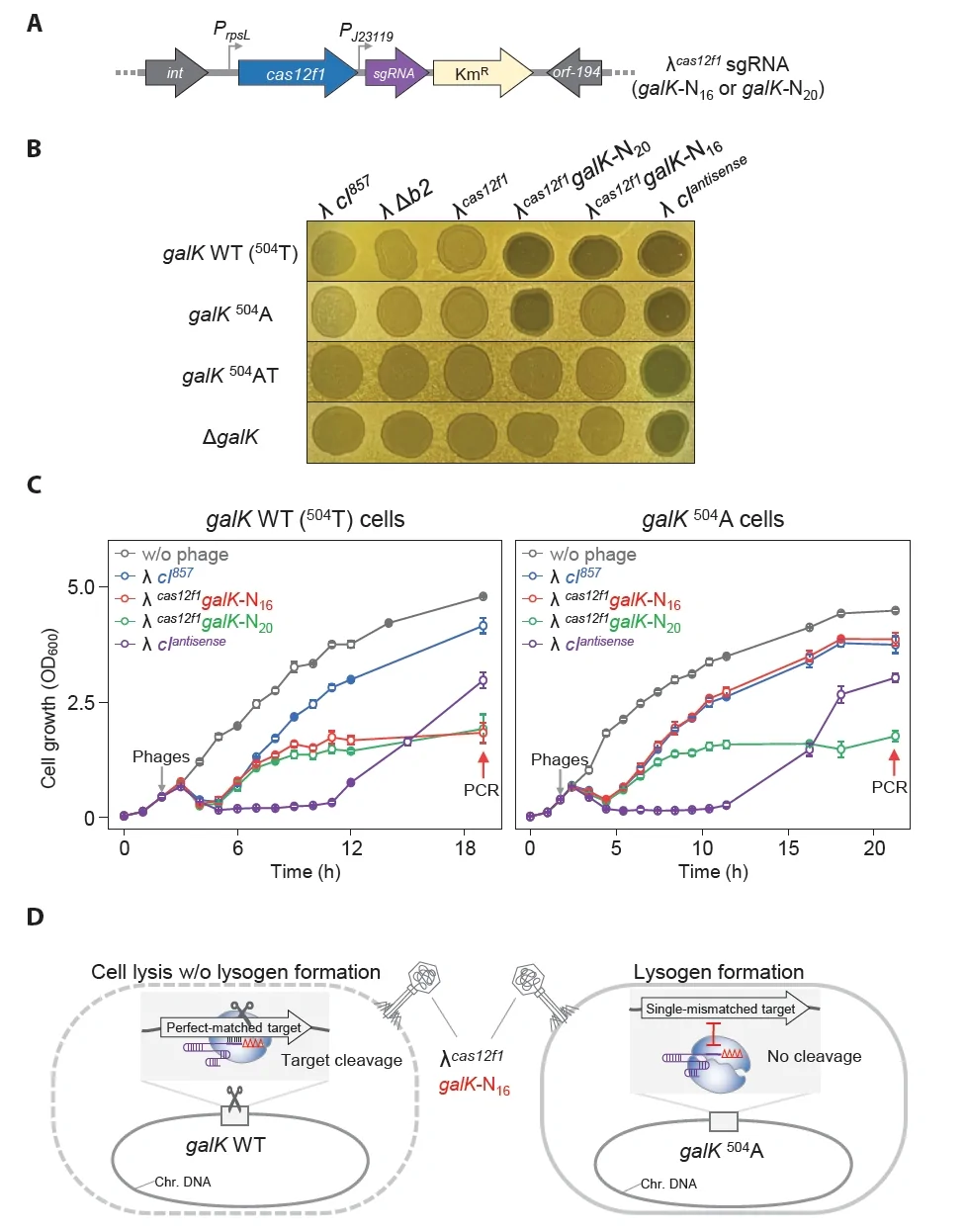
Sequence-specific *galK* target recognition and bacterial cell control using *cas12f1*-*sgRNA*-loaded phages. (A) Construction of synthetic λ phages carrying both the *cas12f1* gene and the *galK*-targeting *sgRNA* gene. Phages with *cas12f1* and *galK* targeting *sgRNA* genes inserted into the λ *cI^857^* genome were constructed. λ*^cas12f1^galK*-N_20_ and λ*^cas12f1^galK*-N_16_ have sgRNA TRS lengths of 20 nt and 16 nt, respectively. (B) Spotting assay of synthetic phages, including *cas12f1*-*sgRNA*-loaded λ phages, on soft agar containing MG1655, MG1655-*galK* ^504^A, MG1655-*galK* ^504^AT, or MG1655-Δ*galK* cells at 30°C for 16 h. (C) Genomic *galK* sequence-specific bacterial control by *cas12f1*-*sgRNA*-loaded phages. The growth of the MG1655 and MG1655-*galK* ^504^A strains at 30°C was measured at OD_600_ after infection with λ*^cas12f1^galK*-N_20_ and λ*^cas12f1^galK*-N_16_ phages. The gray arrow indicates the time point of phage infection. Each OD_600_ measurement represents the average value obtained from three independent cultures. (D) Recognition of single-nucleotide variation and inhibition of lysogeny by λ*^cas12f1^galK*-N_16_ phage. The λ*^cas12f1^galK*-N_16_ phage carries a truncated sgRNA targeting the *galK* WT gene. The Cas12f1-truncated sgRNA complex delivered by the phage cleaves the *galK* WT target through perfect base pairing, thereby preventing lysogeny. However, in *galK* ^504^A cells, the genomic target is not recognized due to the presence of a single mismatch, allowing the formation of lysogenic cells.

We engineered a synthetic phage λ*^cas12f1-sgRNA^* by integrating the genes for *cas12f1* and *galK*-targeting sgRNA into the *b2* region of the bacteriophage λ genome. This synthetic phage was tested for its ability to distinguish single-nucleotide variations in the target sequence of the host genome, and its effects on lysogen formation and cell lysis were elucidated. We constructed λ*^cas12f1^galK*-N_20_ and λ*^cas12f1^galK*-N_16_ with TRS lengths of 20 nt (Δ0) and 16 nt (Δ4), respectively (Fig. 4A). The insertion of *cas12f1* and *sgRNA* genes into the λ *cI^857^* genome was confirmed using PCR, and the base sequence of the sgRNA gene was confirmed through Sanger sequencing (Fig. S4).

For the spotting assay, MG1655 WT cells mixed in soft agar plates were spotted with the engineered phages—λ*^cas12f1^galK*-N_20_, λ*^cas12f1^galK*-N_16_, and λ *cI^antisense^*—and incubated at 30°C for 16 h, forming a clear spot (Fig. 4B). In the soft agar of *galK* ^504^A cells, only the λ*^cas12f1^galK*-N_20_ phage and the λ *cI^antisense^* lytic phage formed clear spots, whereas the λ*^cas12f1^galK*-N_16_ phage formed a turbid spot. This result shows that the phage-loaded Cas12f1-truncated sgRNA (*galK*-N_16_) complex recognized and cleaved the *galK* WT target, preventing lysogen formation. However, λ*^cas12f1^galK*-N_16_ did not recognize and cleave the *galK* ^504^A target.

When cultured at 30°C, the λ *cI^antisense^* lytic phage showed clear spots in *galK* ^504^AT and Δ*galK* host cells. However, λ*^cas12f1^galK*-N_20_ and λ*^cas12f1^galK*-N_16_ formed turbid spots (Fig. 4B). This result indicates that the Cas12f1-sgRNA complex did not affect lysogen formation due to the absence of target sequence variation. All phages formed clear spots in all hosts when cultured at 37°C post-spotting (Fig. S5A).

Next, *galK* WT, *galK* ^504^A, ^504^AT, and Δ*galK* host cells were infected with λ *cI^857^*, λ*^cas12f1^galK*-N_20_, λ*^cas12f1^galK*-N_16_, and λ *cI^antisense^* phages to monitor growth in liquid culture. In *galK* WT cells infected with λ*^cas12f1^galK*-N_20_ and λ*^cas12f1^galK*-N_16_ phages at 30°C, cell growth resumed after lysis, reaching an OD_600_ value of approximately 1.0 (Fig. 4C). However, in *galK* ^504^A cells, only the λ*^cas12f1^galK*-N_20_ phage showed low growth, while cells infected with the λ*^cas12f1^galK*-N_16_ phage reached an OD_600_ value of 3.8, similar to those infected with the λ *cI^857^* phage.

Both streaking of cell cultures grown after lysis due to λ*^cas12f1^galK*-N_20_ and λ*^cas12f1^galK*-N_16_ phage infections in *galK* WT at 30°C and PCR verifications revealed that no lysogens were formed. However, five colonies derived from λ*^cas12f1^galK*-N_16_ infections in *galK* ^504^A cells were confirmed to be lysogens (Fig. S6), consistent with the spotting assay results indicating lysogen formation by λ*^cas12f1^galK*-N_16_ in *galK* ^504^A cells. Infections with λ*^cas12f1^galK*-N_20_ in *galK* ^504^A cells did not result in lysogen formation, as confirmed using colony PCR. In liquid cultures of *galK* ^504^AT and Δ*galK* cells infected with λ*^cas12f1^galK*-N_20_ and λ*^cas12f1^galK*-N_16_ phages, growth curves almost identical to those infected with the λ *cI^857^* phage were observed (Fig. S7). This indicates that the phage-delivered Cas12f1-sgRNA complex did not recognize any target in ^504^AT and Δ*galK* cells.

When monitoring the growth of *galK* WT cells and *galK* ^504^A strains at 37°C, λ*^cas12f1^galK*-N_20_ and λ*^cas12f1^galK*-N_16_ phages displayed growth patterns nearly identical to those of the λ *cI^857^* phage without Cas12f1-sgRNA (Fig. S5B). This similarity is attributed to the inactivation of the heat-labile CI^857^, causing the phages to exclusively undergo the lytic cycle and rendering the presence of Cas12f1-sgRNA irrelevant. These results demonstrate that synthetic phages carrying both *cas12f1* and *sgRNA* genes can effectively recognize and cleave the host cell’s target *galK* gene, suppressing lysogen formation. Moreover, phages carrying truncated sgRNA genes can discern single-nucleotide variations in the target DNA (Fig. 4D).

### Precise control of *E. coli* carrying Shiga toxin genes

Some harmful microbes, such as *Shigella* and *E. coli*, express Shiga toxin, which can be categorized into type 1 and type 2 variants, and the subtype *stx2a* is known to be the most virulent (Fuller et al., 2011). We engineered MG1655 *E. coli* strains harboring the *stx2a* and *stx2g* genes at the *srlAEBD* operon location (Fig. S8). We created phages λ*^cas12f1^stx2a*-152-N_16_ and λ*^cas12f1^stx2a*-218-N_16_, targeting nucleotides 152–167 (16 nt) and 218–233 (16 nt) of the *stx2a* gene, respectively (Fig. S9). Next, we investigated the efficacy of these phages carrying truncated sgRNAs and the Cas12f1 system targeting the *stx2a* gene in inducing lysis and preventing lysogeny in MG1655 *E. coli* strains with different *stx2* gene subtypes.

**Fig. 5.**
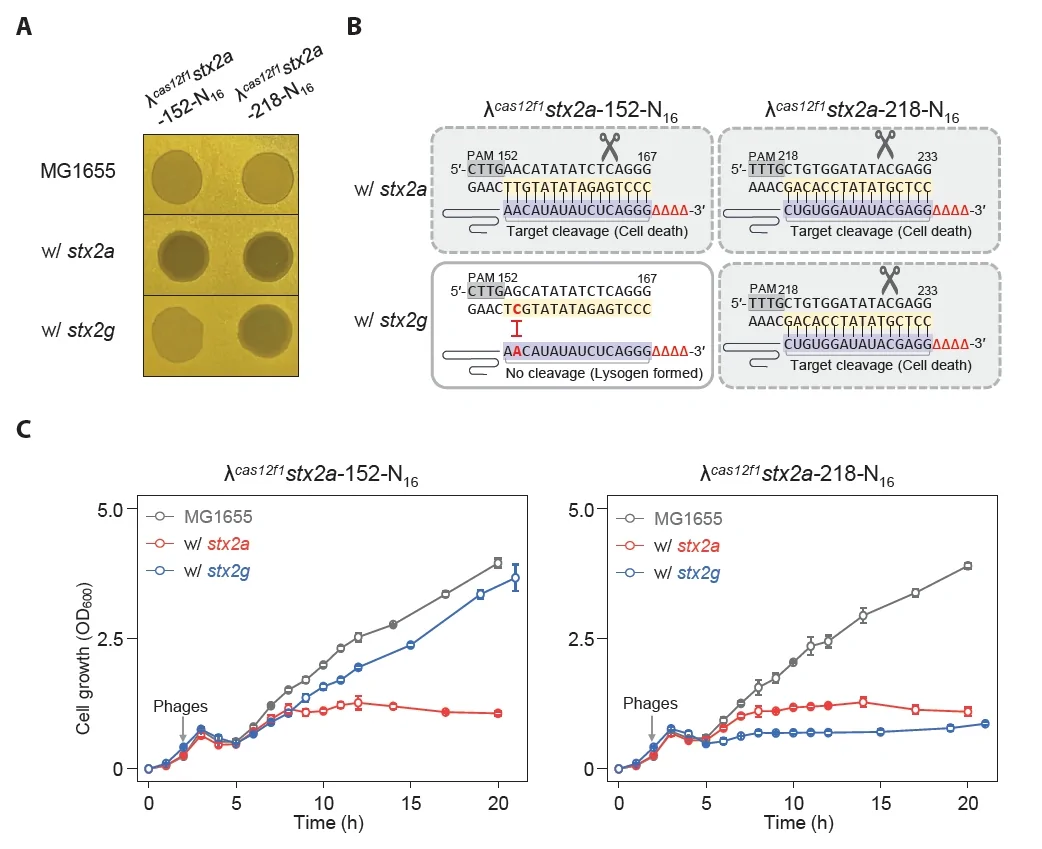
Controlled lysis of *E. coli* carrying different Shiga toxin gene subtypes using *cas12f1*-*sgRNA*-loaded phages. (A) Spotting assay of synthetic λ*^cas12f1^stx2a*-152-N_16_ and λ*^cas12f1^stx2a*-218-N_16_ phages on soft agar containing various *E. coli* cells carrying *stx2* subtypes. *stx2a* and *stx2g* genes were inserted into the *srl* operon of the MG1655 genome. (B) Target nucleotide sequences of *stx2* gene subtypes recognized by truncated sgRNAs (N_16_) of the CRISPR-Cas12f1 system. Red nucleotides represent single-nucleotide variations specific to the *stx2* subtypes that prevent perfect base-pairing with the *sgRNA* sequence. (C) Genomic *stx2* subtype sequence-specific bacterial control using *cas12f1*-*sgRNA*-loaded phages. The growth of MG1655 strains carrying *stx2a* and *stx2g* genes at 30°C was monitored after infection with synthetic λ*^cas12f1^stx2a*-152-N_16_ and λ*^cas12f1^stx2a*-218-N_16_ phages. The gray arrow indicates the time point of phage infection. Each OD_600_ measurement represents the average value obtained from three independent cultures.

The spotting assay results showed that the λ*^cas12f1^stx2a*-152-N_16_ phage formed clear spots exclusively in strains carrying the *stx2a* gene, while the λ*^cas12f1^stx2a*-218-N_16_ phage formed clear spots in both *stx2a* and *stx2g* gene-carrying strains (Fig. 5A). This indicates that the λ*^cas12f1^stx2a*-152-N_16_ phage did not recognize targets in the *stx2g* gene due to a single-nucleotide mismatch, thus allowing lysogen formation, as evidenced by turbid spots (Fig. 5B).

In flask culture, consistent with the spotting assay results, the λ*^cas12f1^stx2a*-152-N_16_ phage inhibited the growth of strains carrying the *stx2a* gene, while strains with the *stx2g* gene exhibited growth curves similar to those of strains infected with the λ *cI^857^* phage (Fig. 5C and Fig. S10). However, the λ*^cas12f1^stx2a*-218-N_16_ phage inhibited the growth of both *stx2a* and *stx2g* gene-carrying strains due to perfect base paring between the target DNA and truncated sgRNA.

Additionally, when phages were not introduced or when infected with the λ *cI^857^* phage, the same growth patterns were observed in all four types of strains (Fig. S10). These results demonstrate that synthetic λ phages carrying *cas12f1* and truncated *sgRNA* can specifically control subtypes of the *stx2* pathogenic genes in the host’s genome by distinguishing single-nucleotide variations.

## Discussion

Prophages integrate into the host chromosome, facilitating horizontal gene transfer of antibiotic-resistance genes, pathogenic determinants, and other genetic elements (Wendling et al., 2021). When the temperate phage λ infects *E. coli*, cell growth is observed after lysis of the host cells, a phenomenon attributed to the formation of lysogens (Maynard et al., 2010, 2012; Sinha et al., 2017). Consistent with previous studies, infection of *E. coli* MG1655 cells cultured at 30°C with λ *cI^857^* and λ Δ*b2* resulted in the formation of turbid spots on soft agar (Fig. 1A) and an increase in OD_600_ after cell lysis in liquid culture (Fig. 1B). Lambdoid prophages provide superinfection immunity to their hosts, preventing further phage infections (Fogg et al., 2010). PCR experiments confirmed that cells exhibiting post-infection growth without lysis were indeed lysogenic (Fig. S1). The formation of host cell lysogens by prophages poses a challenge to the use of phages in bacterial control strategies (Bondy-Denomy et al., 2016).

In this study, we first engineered a bacteriophage λ lacking the *b2* region of its genome and equipped with the Cas12f1 nuclease. Subsequently, we characterized the λ*^cas12f1^* phage by infecting cells carrying sgRNA plasmids (Fig. 2A & 2B). The λ*^cas12f1^* phage effectively infected the host and formed clear spots only in the presence of the sgRNA. This observation highlighted the capability of the Cas12f1-sgRNA complex to inhibit lysogen formation in the host (Fig. 2C). A similar inhibitory effect on lysogeny has been reported previously with a *C. difficile*-specific temperate phage equipped with the CRISPR-Cas3 system (Selle et al., 2020).

Cells lacking the *galK* gene (Δ*galK*) and those with the *galK* ^504^AT variant, which had two mismatches between the genomic *galK* target and sgRNA, did not facilitate targeting by the Cas12f1 nuclease-sgRNA complex, resulting in the formation of turbid spots indicative of lysogen formation. Conversely, cells with the *galK* ^504^A variant, which had only a single mismatch, were recognized as targets like *galK* WT (^504^T) cells, resulting in clear spot formation (Fig. 2B). This finding highlights a notable drawback of the CRISPR-Cas system, specifically its mismatch tolerance, where targeting occurs despite the presence of a few mismatches between the target DNA and sgRNA (Huang et al., 2022). A similar issue with Cas9 has been solved by using truncated sgRNA, which accurately identifies single-nucleotide mismatches, a phenomenon referred to as mismatch intolerance (Lee et al., 2021). Furthermore, precise genome editing has been demonstrated in the Cas12f1 system using truncated sgRNA, enabling the effective discrimination of single-nucleotide variations (Lee et al., 2023).

To evaluate and address the issue of mismatch tolerance (Fig. 2B), we performed a spotting assay using the λ*^cas12f1^* phage to infect *galK* WT (^504^T) and *galK* ^504^A cells, each carrying plasmids with truncated sgRNAs of various lengths (Lee et al., 2023). The results demonstrated that the λ*^cas12f1^* phage could effectively distinguish single-nucleotide variations in the host genome. It caused lysis in *galK* WT cells but allowed lysogen formation in *galK* ^504^A, where the Cas12f1-N_16_ sgRNA failed to recognize the target sequence (Fig. 3A). This finding underscores the capability of phage-delivered Cas12f1 to discern single-nucleotide variations within sgRNA target sequences in the host genome.

However, since the sgRNA for Cas12f1 was introduced into the host via multiple-copy plasmids, we further investigated whether single-copy sgRNA integrated into the phage genome could still effectively distinguish single-nucleotide variations. The results demonstrated that the λ*^cas12f1^galK*-N_16_ phage, which carried both the sgRNA and Cas12f1, effectively killed *galK* WT cells and suppressed lysogen formation but did not kill *galK* ^504^A cells (Fig. 4A & 4B), allowing lysogen formation (Fig. S6). Thus, the λ*^cas12f1^galK*-N_16_ phage carrying truncated sgRNA and Cas12f1 was confirmed to be capable of distinguishing single-nucleotide variations in the genome (Fig. 4B). Additionally, in liquid culture, while the λ*^cas12f1^galK*-N_16_ phage-infected *galK* WT host cells initially exhibited slight growth after lysis, their growth was notably suppressed compared to the λ *cI^antisense^* lytic phage-infected host bacteria at 18 h (Fig. 4C).

After infection with the λ*^cas12f1^galK*-N_16_ phage, *galK* ^504^A cells exhibited growth but were confirmed to be lysogens using PCR, indicating that the phage-delivered Cas12f1-truncated sgRNA complex did not recognize *galK* ^504^A as a target (Fig. S6). Conversely, as shown in Fig. 1B, cells infected with the lytic λ *cI^antisense^* phage also regrew but did not become lysogens (Fig. S1B), likely due to the emergence of phage-resistant mutants. Figure 4C illustrates that cell growth at 18 h post-infection was more suppressed by λ*^cas12f1^galK*-N_20_ or λ*^cas12f1^galK*-N_16_ phages than by the λ *cI^antisense^* phage, suggesting that the phage-delivered Cas12f1-sgRNA complex effectively eliminated the emerged phage-resistant mutants. Additionally, the observation of residual cell growth post-lysis by the Cas12f1-loaded λ phage, despite the absence of lysogeny, could be attributed to various factors, including the elimination, modification, or masking of phage receptors (Egido et al., 2022), emergence of persisters (Mamontov et al., 2022) and mutations in the PAM or protospacer (Schelling et al., 2023). Indeed, previous studies using the M13 phagemid to deliver the Cas9 system have reported observing escape cells due to mutations in the spacer, tracrRNA, or target site (Lam et al., 2021).

When the λ*^cas12f1^stx2a*-152-N_16_ phage infected *E. coli* strains carrying different subtypes of the *stx2* gene, specifically *stx2a* and *stx2g*, it could distinguish single-nucleotide variations between the two *stx2* gene subtypes, effectively lysing cells with the *stx2a* gene (Fig. 5C). This distinction is crucial given that *stx2* is known to vary in virulence depending on the subtype, with *stx2a* being the most virulent (Fuller et al., 2011). Such selective control over gene subtypes enhances precision in targeting specific harmful microbes within a microbial population.

## Conclusion

The CRISPR-Cas system, which originally evolved as part of the microbial immune system against foreign nucleic acids from phages, is now finding applications in diverse fields, including diagnostics (Chakraborty et al., 2022) and therapeutics (Luthra et al., 2021). However, the phenomenon of mismatch tolerance has posed challenges in its applications in genome editing, diagnostics, and therapeutics (Mengstie et al., 2024). In this study, we introduced a CRISPR-Cas-loaded phage designed to detect target sequence variations with single-nucleotide precision, demonstrated within the *E. coli*-λ model. While comprehensive evaluation in complex environments, such as microbial communities and animal models, remains necessary, our investigation into synthetic phages represents a significant step forward in the development of precise CRISPR-Cas-based antimicrobial agents.

## Supplementary Information

Table S1. Strains, phages and plasmids used in this study

Table S2. Primers used in this study

Fig. S1. Confirmation of λ lysogen formation using PCR.

Fig. S2. Sanger sequences of the *galK* mutant strains.

Fig. S3. Spotting assays of various λ phages on *galK* mutant cells carrying various 3ʹ-end truncated sgRNA plasmids at 37℃.

Fig. S4. Construction of λ^cas12f1^ galK-N20 and λ^cas12f1^ *galK*-N_16_ phages.

Fig. S5. Growth of *E. coli* *galK* mutant cells infected with various λ phages, including λ^cas12f1^ *galK*-N_20_ and λ^cas12f1^ *galK*-N_16_, at 37℃.

Fig. S6. Confirmation of lysogen formation in *galK* WT and *galK*^504^A cells infected with λ^cas12f1^ *galK*-N_20_ or λ^cas12f1^*galK*-N_16_ phages at 30°C and 37°C.

Fig. S7. Growth curves of *galK* ^504^AT and Δ*galK* cells infected with λ cI^857^, λ^cas12f1^ *galK*-N_20_, or λ^cas12f1^ *galK*-N_16_ phages at 30°C.

Fig. S8. Sanger sequences of *E. coli* strains carrying different *stx2* gene subtypes.

Fig. S9. Construction of λ^cas12f1^*stx2a*-152-N_16_ and λ^cas12f1^*stx2a*-218-N_16_ phages.

Fig. S10. Growth curves of λ cI^857^ phage-infected *E. coli* cells carrying the *stx2a* or *stx2g* gene at 30°C. The gray arrows indicate the time points of phage infection.
