# Supplementary material for: Single nucleotide genome recognition and selective bacterial lysis using synthetic phages loaded with CRISPR-Cas12f1-truncated sgRNA
Source: J Microbiol. 2025 Feb 27;63(2):e2501012. doi: 10.71150/jm.2501012 (PMC13577792; doi:10.71150/jm.2501012)
Supplement: Supplementary file 2 [file jm-2501012-Supplementary-Fig-S10.pdf]

Fig. S10

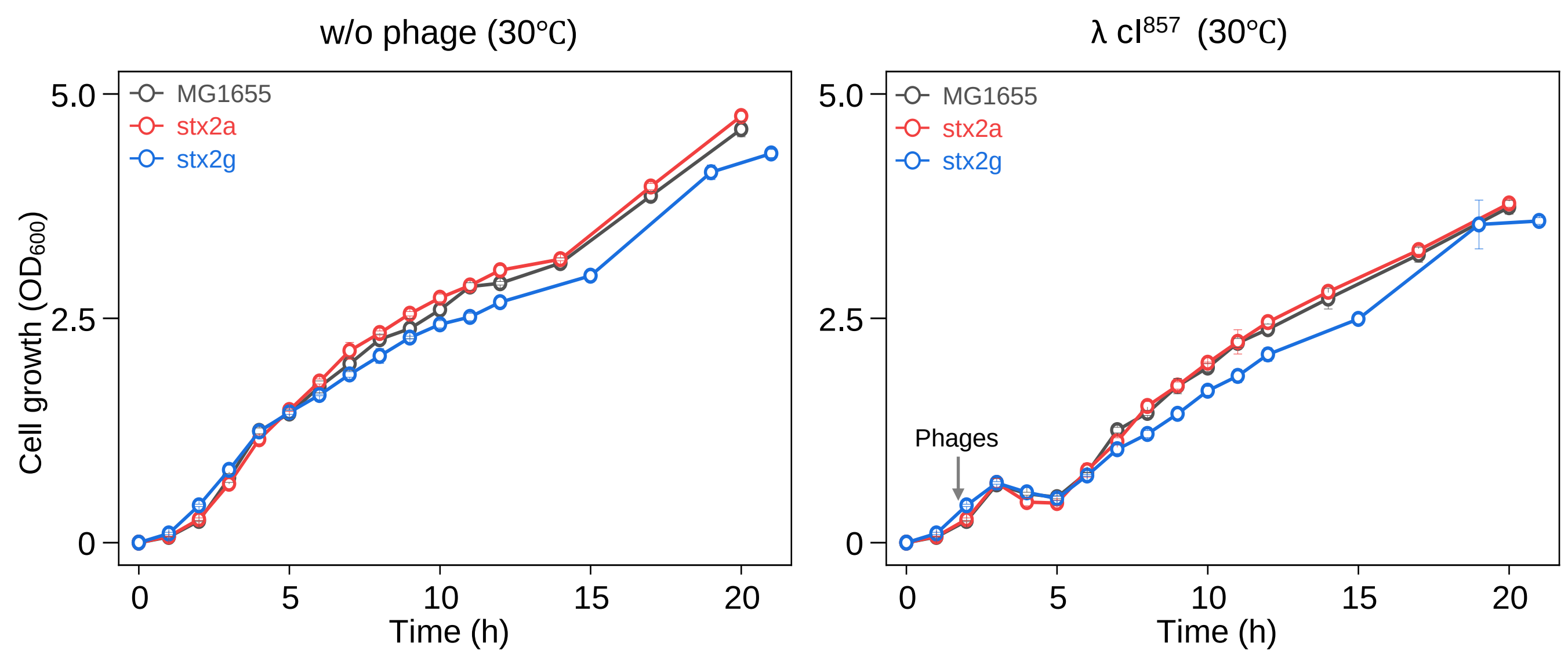

Fig. S10. Growth curves of λ *cI*^{857} phage-infected *E. coli* cells carrying the *stx2a* or *stx2g* gene at 30°C. The gray arrows indicate the time points of phage infection.
