# Supplementary material for: Single nucleotide genome recognition and selective bacterial lysis using synthetic phages loaded with CRISPR-Cas12f1-truncated sgRNA
Source: J Microbiol. 2025 Feb 27;63(2):e2501012. doi: 10.71150/jm.2501012 (PMC13577792; doi:10.71150/jm.2501012)
Supplement: Supplementary file 3 [file jm-2501012-Supplementary-Fig-S2.pdf]

Fig. S2

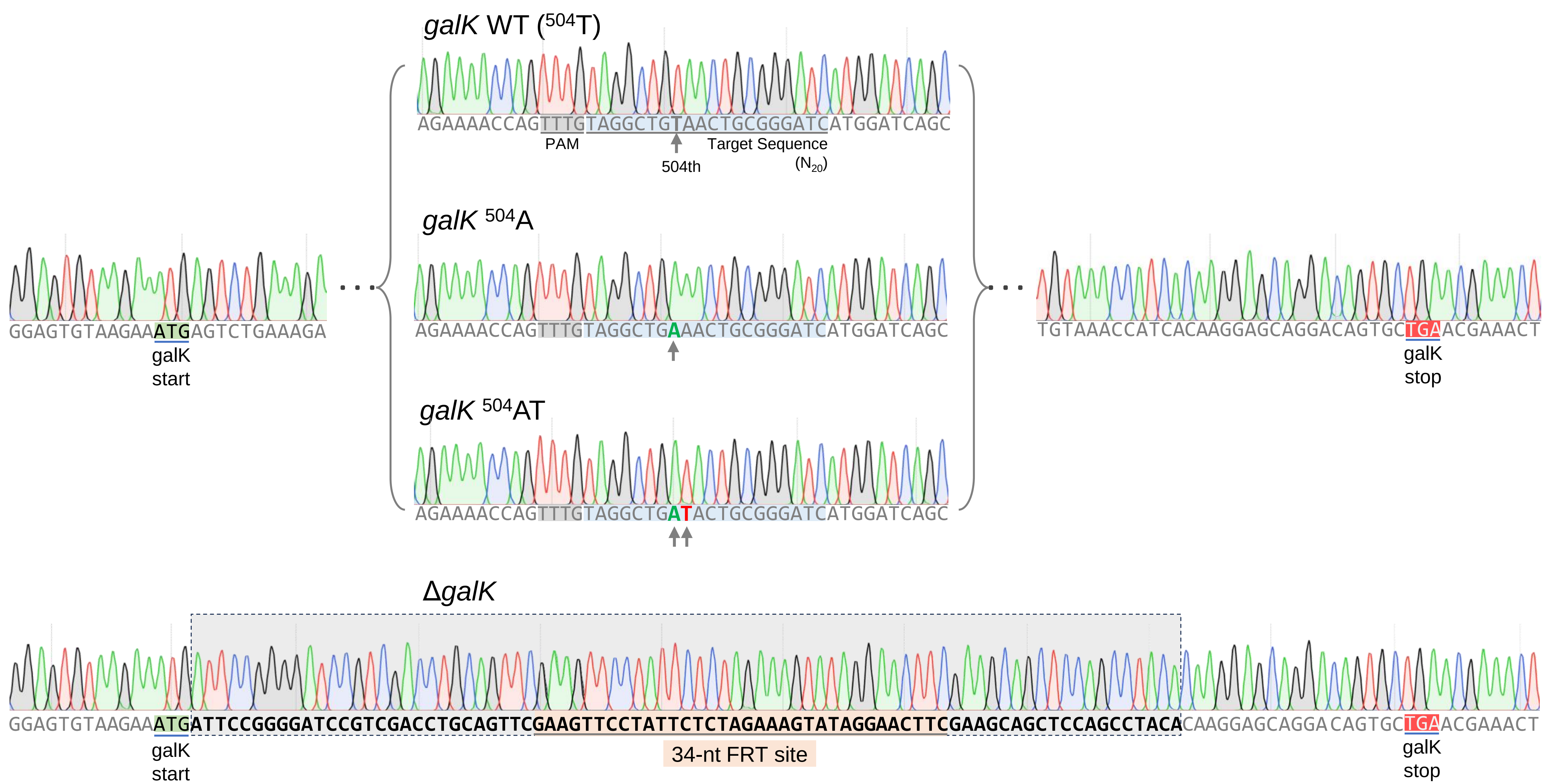

Fig. S2. Sanger sequences of the *galK* mutant strains.

The Sanger sequence confirming the genotype of the *galK* mutant strains is shown. The gray arrows indicate the ^{504}T, ^{504}A, and ^{504}AT variations in the *galK* gene. The gray-shaded region represents the part of the *galK* gene that has been deleted and replaced with a 34-nt FRT site.
