# Supplementary material for: Single nucleotide genome recognition and selective bacterial lysis using synthetic phages loaded with CRISPR-Cas12f1-truncated sgRNA
Source: J Microbiol. 2025 Feb 27;63(2):e2501012. doi: 10.71150/jm.2501012 (PMC13577792; doi:10.71150/jm.2501012)
Supplement: Supplementary file 4 [file jm-2501012-Supplementary-Fig-S3.pdf]

Fig. S3

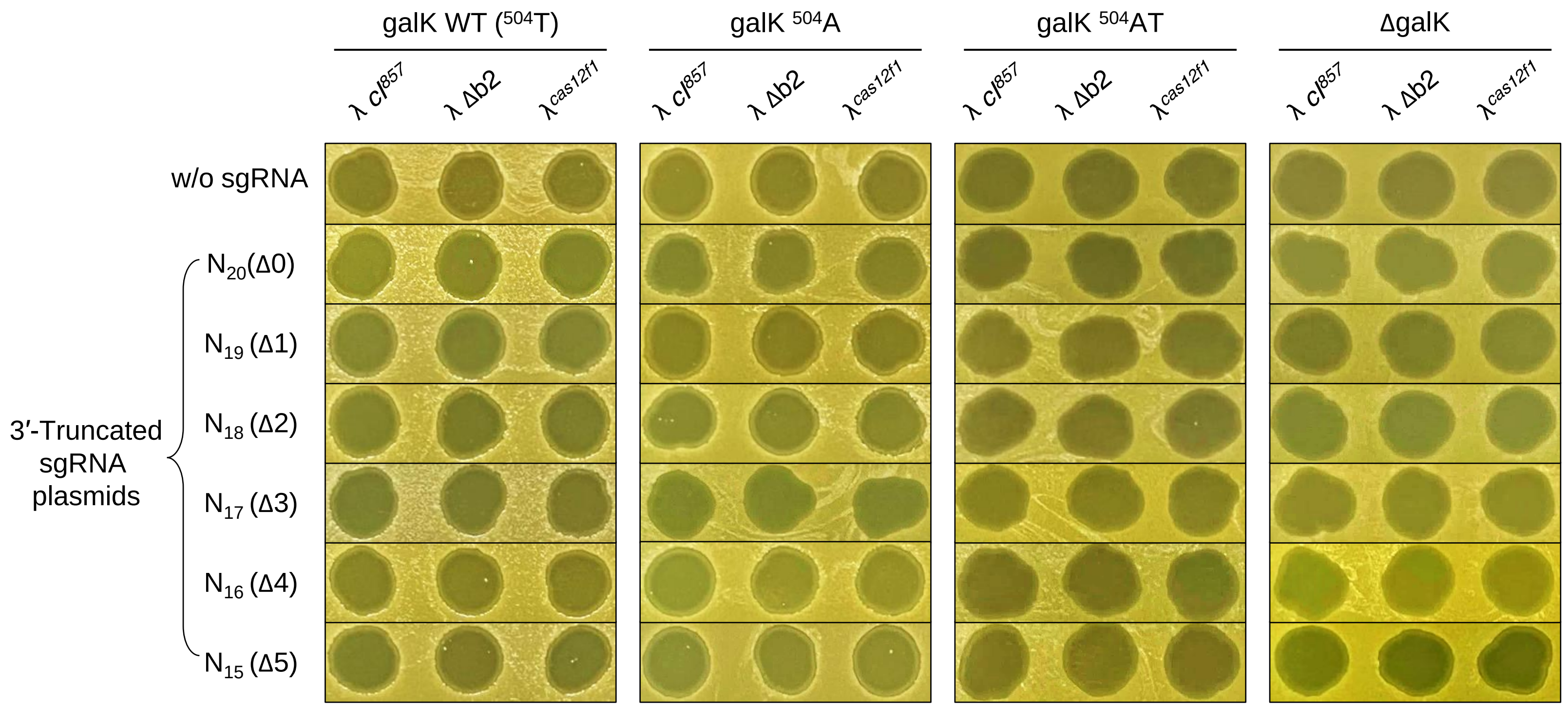

Fig. S3. Spotting assays of various λ phages on *galK* mutant cells carrying various 3'-end truncated sgRNA plasmids at 37℃.

λ cI^{857}, λ Δ*b2*, and $\lambda^{cas12f1}$ phages were spotted on soft agar with ^{504}T, ^{504}A, ^{504}AT, and Δ*galK* cells. N_{20}– N_{15} indicates the nucleotide length of the sgRNA target recognition sequence (TRS). Δ0–Δ5 denotes the number of nucleotides truncated from the 3'-end of the sgRNA.
