# Supplementary material for: Single nucleotide genome recognition and selective bacterial lysis using synthetic phages loaded with CRISPR-Cas12f1-truncated sgRNA
Source: J Microbiol. 2025 Feb 27;63(2):e2501012. doi: 10.71150/jm.2501012 (PMC13577792; doi:10.71150/jm.2501012)
Supplement: Supplementary file 5 [file jm-2501012-Supplementary-Fig-S4.pdf]

Fig. S4

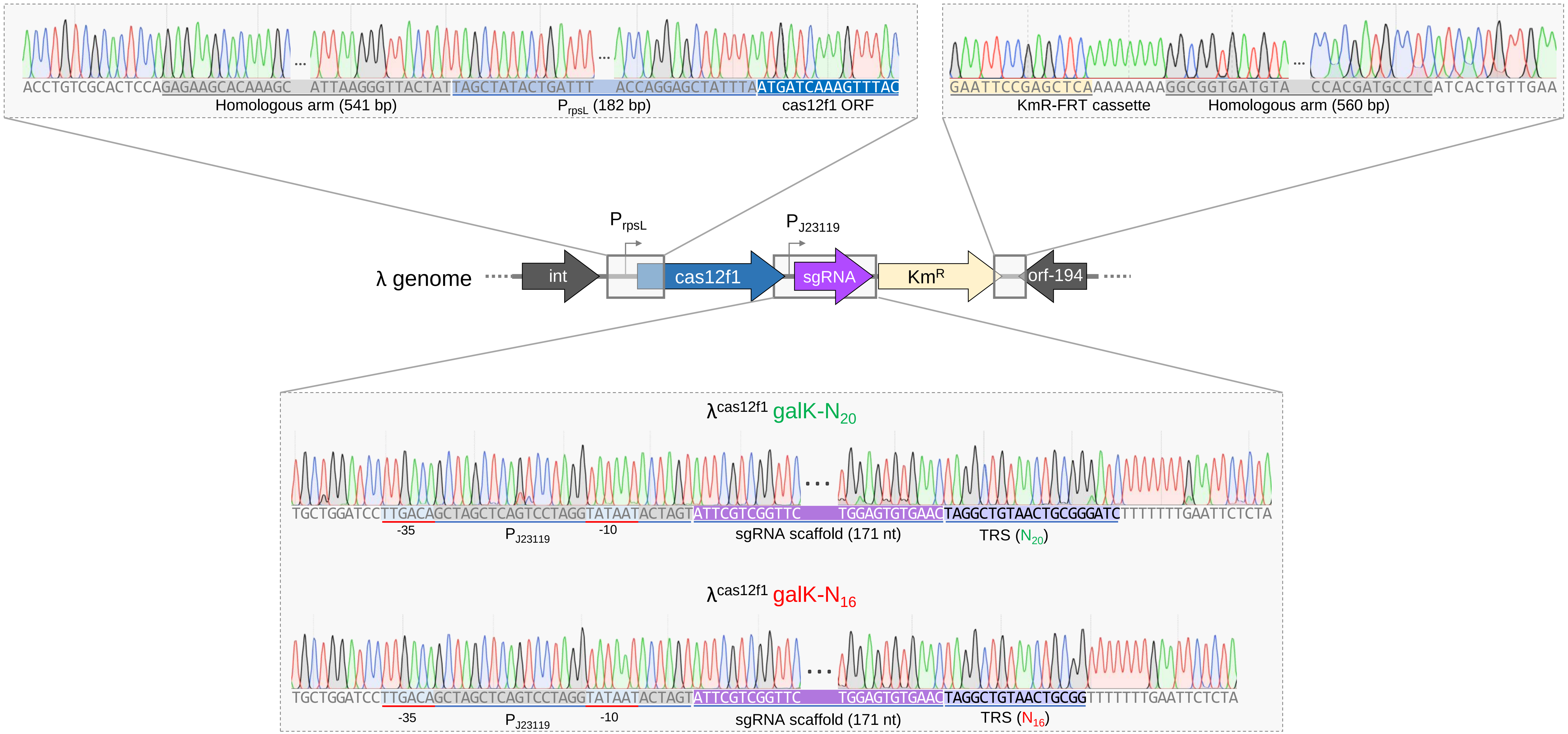

Fig. S4. Construction of $\lambda^{cas12f1}$ *galK*-N_{20} and $\lambda^{cas12f1}$ *galK*-N_{16} phages.

The *cas12f1* gene and *galK*-targeting sgRNA (*galK*-N_{20} or *galK*-N_{16}) gene were inserted in the *b2* region of the λ cI^{857} phage. *galK*-N_{20} and *galK*-N_{16} represent two different truncated sgRNAs with *galK* TRS of 20 nucleotides and 16 nucleotides, respectively.
