# Supplementary material for: Single nucleotide genome recognition and selective bacterial lysis using synthetic phages loaded with CRISPR-Cas12f1-truncated sgRNA
Source: J Microbiol. 2025 Feb 27;63(2):e2501012. doi: 10.71150/jm.2501012 (PMC13577792; doi:10.71150/jm.2501012)
Supplement: Supplementary file 6 [file jm-2501012-Supplementary-Fig-S5.pdf]

Fig. S5

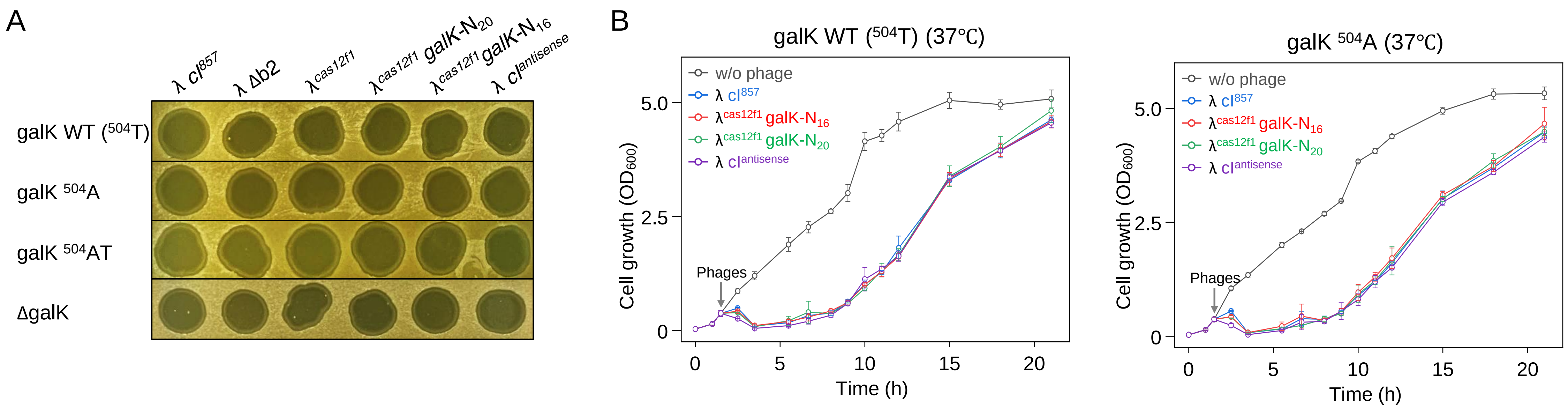

Fig. S5. Growth of *E. coli galK* mutant cells infected with various λ phages, including $\lambda^{cas12f1}$ *galK*-N_{20} and $\lambda^{cas12f1}$ *galK*-N_{16}, at 37℃.

(A) Spotting assays of various λ phages on soft agar with *E. coli galK* mutants at 37℃.

(B) Growth curves of *galK* WT and *galK* ^{504}A cells infected with various phages, including $\lambda^{cas12f1}$ *galK*-N_{20} and $\lambda^{cas12f1}$ *galK*-N_{16}. The gray arrows indicate the time points of phage infection.
