# Supplementary material for: Single nucleotide genome recognition and selective bacterial lysis using synthetic phages loaded with CRISPR-Cas12f1-truncated sgRNA
Source: J Microbiol. 2025 Feb 27;63(2):e2501012. doi: 10.71150/jm.2501012 (PMC13577792; doi:10.71150/jm.2501012)
Supplement: Supplementary file 7 [file jm-2501012-Supplementary-Fig-S6.pdf]

Fig. S6

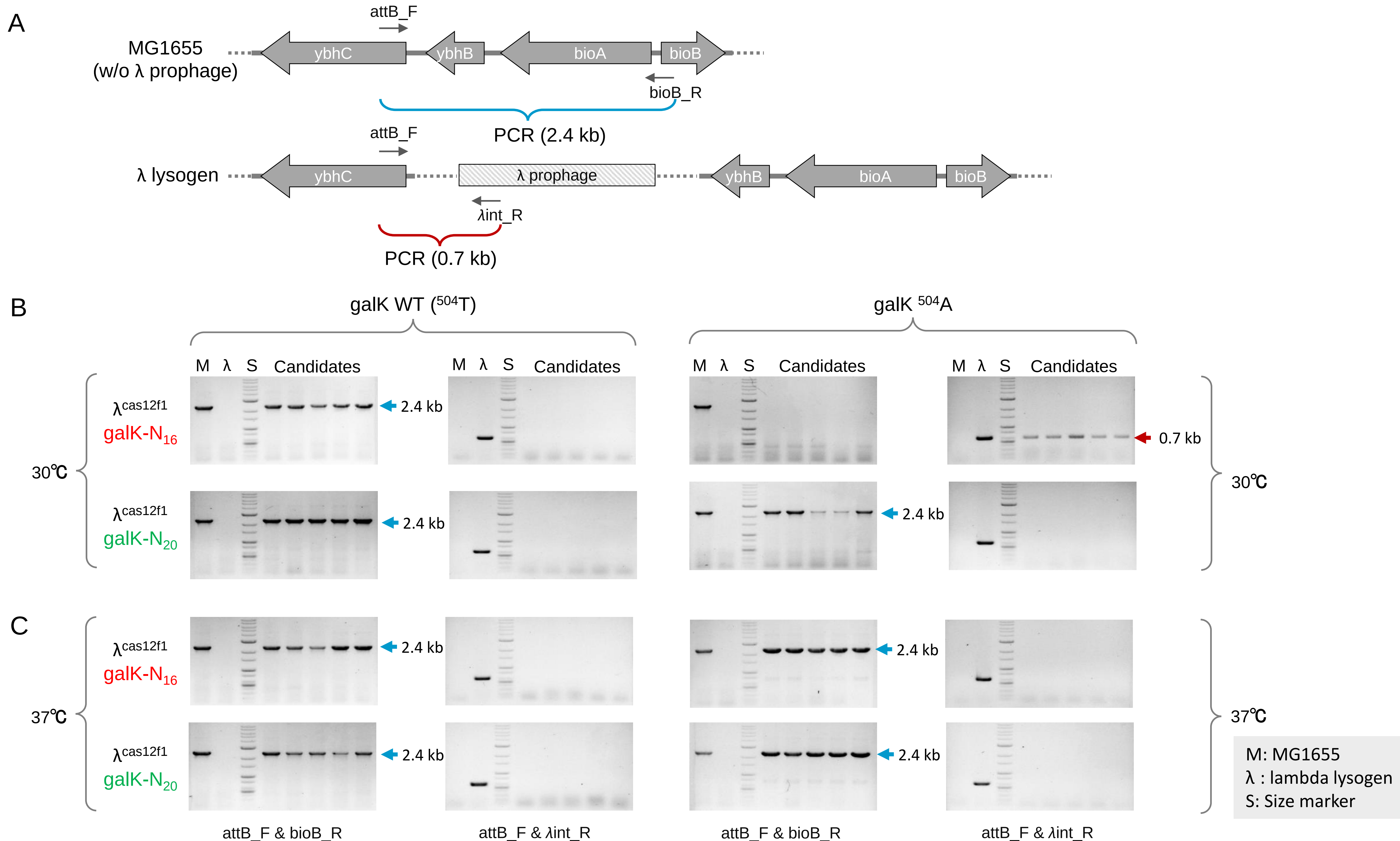

Fig. S6. Confirmation of lysogen formation in *galK* WT and *galK* ^{504}A cells infected with λ^{cas12f1} *galK*-N$_{20}$ or λ^{cas12f1}*galK*-N$_{16}$ phages at 30°C and 37°C.

(A) Locations of PCR primers on the genome that distinguish between *E. coli* MG1655 and λ lysogenic MG1655 cells. The gray arrows represent the PCR primers, and the sizes of the PCR products formed by each primer are indicated.

(B) Confirmation of λ lysogen formation using PCR in colonies obtained by spreading cultures of *E. coli galK* WT and *galK* ^{504}A cells, which were infected with λ^{cas12f1} *galK*-N$_{20}$ or λ^{cas12f1}*galK*-N$_{16}$ phages and regrown at both 30°C and 37°C. Blue arrows indicate the absence of λ prophage in the *E. coli* genome, while red arrows signify the presence of λ prophage in the genome. Five colonies were selected in each case, and PCR reactions using two primer pairs (*attB_F* + *bioB_R* and *attB_F* + *λint_R*) were performed for each colony.
