# Supplementary material for: Single nucleotide genome recognition and selective bacterial lysis using synthetic phages loaded with CRISPR-Cas12f1-truncated sgRNA
Source: J Microbiol. 2025 Feb 27;63(2):e2501012. doi: 10.71150/jm.2501012 (PMC13577792; doi:10.71150/jm.2501012)
Supplement: Supplementary file 8 [file jm-2501012-Supplementary-Fig-S7.pdf]

Fig. S7

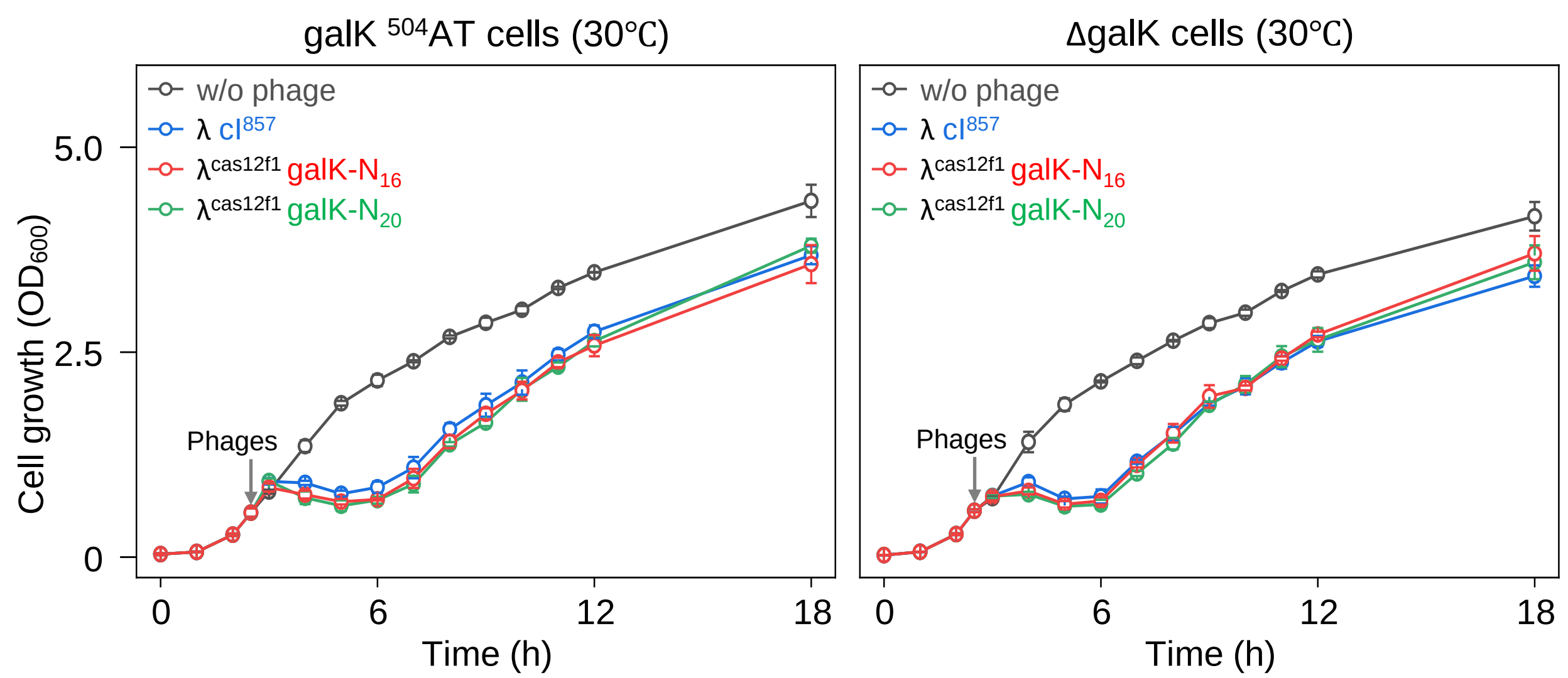

Fig. S7. Growth curves of *galK* 504AT and Δ*galK* cells infected with λ cI^{857}, $\lambda^{cas12f1}$ *galK*-N_{20}, or $\lambda^{cas12f1}$ *galK*-N_{16} phages at 30°C.

The gray arrows indicate the time points of phage infection.
