# Supplementary material for: Single nucleotide genome recognition and selective bacterial lysis using synthetic phages loaded with CRISPR-Cas12f1-truncated sgRNA
Source: J Microbiol. 2025 Feb 27;63(2):e2501012. doi: 10.71150/jm.2501012 (PMC13577792; doi:10.71150/jm.2501012)
Supplement: Supplementary file 9 [file jm-2501012-Supplementary-Fig-S8.pdf]

Fig. S8

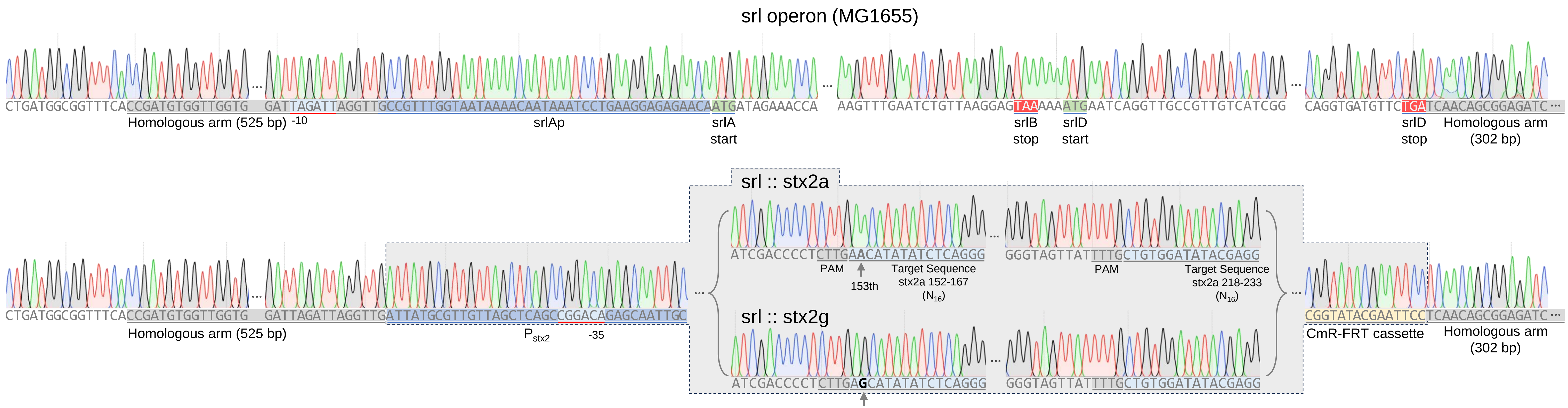

Fig. S8. Sanger sequences of *E. coli* strains carrying different *stx2* gene subtypes.

The Sanger sequence of the *stx2a* and *stx2g* genotypes inserted into the *srl* operon is shown. The gray region indicates parts of the sequences (157–167 and 218–233) of the *stx2a* and *stx2g* genes inserted into the *srl* operon. The gray arrows indicate the nucleotide variations observed in the subtype genes.
