# Supplementary material for: Single nucleotide genome recognition and selective bacterial lysis using synthetic phages loaded with CRISPR-Cas12f1-truncated sgRNA
Source: J Microbiol. 2025 Feb 27;63(2):e2501012. doi: 10.71150/jm.2501012 (PMC13577792; doi:10.71150/jm.2501012)
Supplement: Supplementary file 10 [file jm-2501012-Supplementary-Fig-S9.pdf]

**Fig. S9**

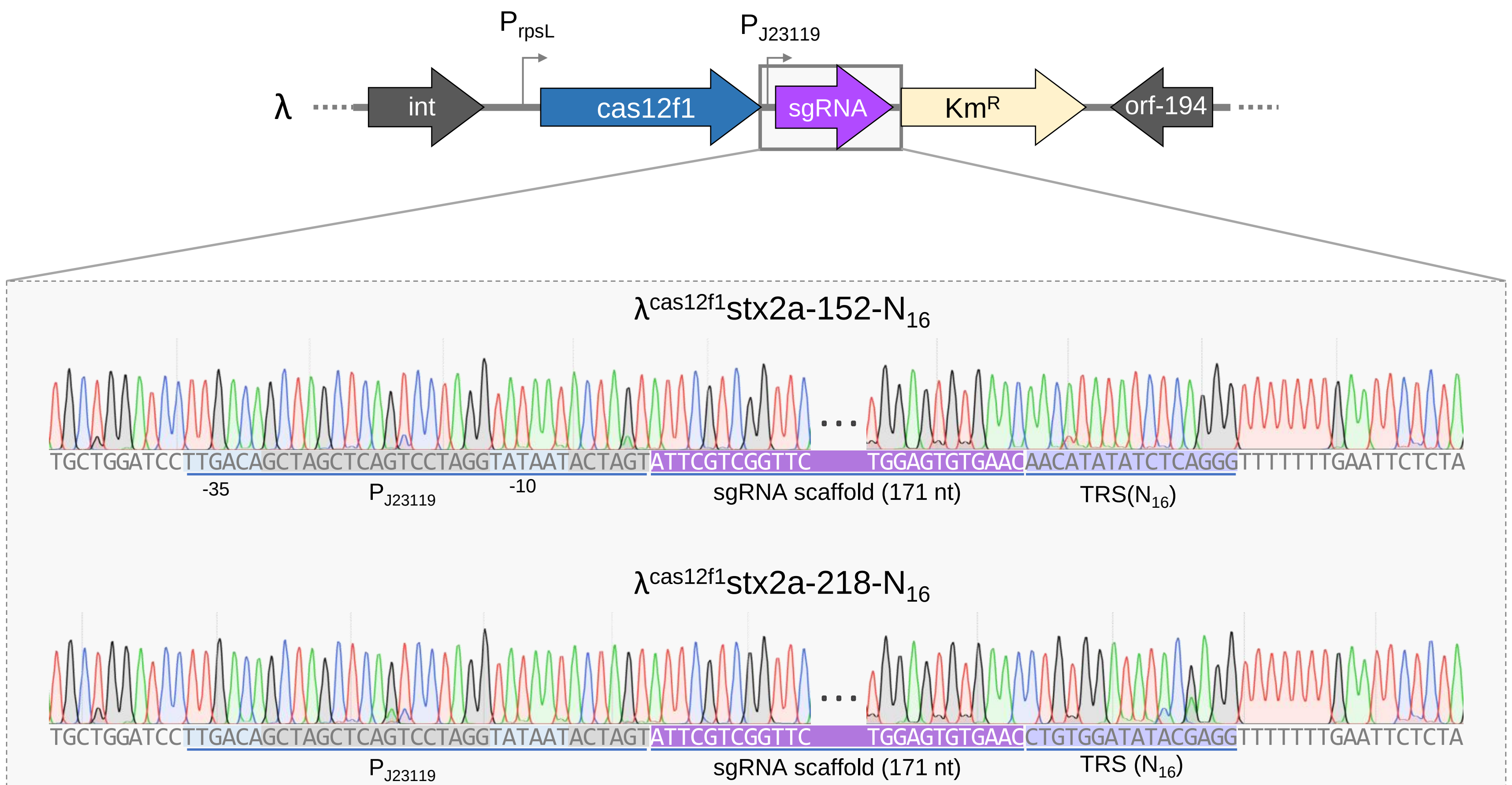


**Fig S9.** Construction of λ$^{cas12f1}$*stx2a-152*-N$_{16}$ and λ$^{cas12f1}$*stx2a-218*-N$_{16}$ phages.

The Sanger sequence of the promoter and target recognition sequence of the 3'-end truncated sgRNA in the phage genome is shown.
